# Association between dairy consumption and cardiovascular disease events, bone fracture and all-cause mortality

**DOI:** 10.1371/journal.pone.0271168

**Published:** 2022-09-09

**Authors:** Jing Guo, David I. Givens, Berit Lilienthal Heitmann

**Affiliations:** 1 Institute for Food, Nutrition and Health (JG and DIG), University of Reading, Reading, United Kingdom; 2 School of Food Science and Nutrition, Faculty of Environment, University of Leeds, Leeds, United Kingdom; 3 Research Unit for Dietary Studies, The Parker Institute, Bispebjerg and Frederiksberg Hospital, Copenhagen, Denmark; 4 Center for Clinical Research and Prevention, Bispebjerg and Frederiksberg Hospital, Copenhagen, Denmark; 5 The Boden Institute of Obesity, Nutrition, Exercise & Eating Disorders, The University of Sydney, Sydney, Australia; 6 Department of Public Health, Section for General Practice, University of Copenhagen, Copenhagen, Denmark; Hualien Tzu Chi Hospital Buddhist Tzu Chi Medical Foundation, TAIWAN

## Abstract

Dairy products are important constituents of a healthy and balanced diet, but their association with health outcomes remains to be established. We investigated the association of total dairy, total fermented dairy, and different dairy subtypes (including total/high-fat/low-fat milk, yogurt, cheese, butter, and cream) and the risk of cardiovascular disease (CVD), coronary heart disease (CHD), bone fracture and all-cause mortality among 1746 Danish healthy men and women (30–60 years, 52%female). Hazard ratios (HRs) and 95% CIs were estimated using the multivariable Cox proportional hazard models. During a mean follow-up of 30 years, incident cases of CVD (*n* = 904), CHD (*n* = 332), fracture (*n* = 447) and all-cause mortality (*n* = 680) were reported. High intake of total fermented dairy was associated with lower fracture risk (HR 0.67, 95% CI: 0.51–0.90, *P* = 0.02) than observed in the lowest tertile of the fermented dairy group. Furthermore, high intake of low-fat milk was associated with lower risks of CVD (HR 0.84, 95% CI: 0.68–1.03, *P* = 0.03), CHD (HR 0.82, 95% CI: 0.59–1.16, *P* = 0.04), and all-cause mortality (HR 0.77, 95% CI: 0.61–0.97, *P* = 0.004) compared with the lowest tertile of low-fat milk group. No associations were found with other dairy subtypes. The findings from this prospective cohort study suggest an inverse association between total fermented dairy and fracture risk, and also inverse associations were found between low-fat milk consumption and risk of CVD, CHD and all-cause mortality.

## Introduction

Dairy products play an important part in a healthy and balanced diet, as these foods are naturally enriched with protein, several vitamins and minerals [1]. However, many dairy products are also high in saturated fats which might have adverse effects on cardiometabolic health [2]. The available evidence was summarised by two recent meta-analyses [3, 4], which showed neutral or beneficial associations between dairy products and the risk of cardiovascular disease (CVD) and all-cause mortality. However, more detailed data on different types of dairy consumption and CVD or all-cause mortality are needed. Furthermore, observational data on the association of different types of dairy consumption and bone fracture risk have shown inconclusive results [5–7]. The recent meta-analysis [6] summarised the available cohort studies and concluded that yogurt and cheese were inversely associated with risk of hip fracture but not total dairy products and cream. There was insufficient evidence from the cohort studies on the association between milk consumption and hip fracture risk. Therefore, we examined the association of total dairy consumption, total fermented dairy, and dairy subtypes (including total/high-fat/low-fat milk, yogurt, cheese, butter, and cream) with cardiovascular disease, coronary heart disease (CHD), fracture and all-cause mortality by using the WHO-initiated survey Monitoring of Trends and Determinants in Cardiovascular Diseases (MONICA) cohort.

## Subjects and methods

### Study populations

The MONICA-I cohort (1982–1984) in the current study originates from the Danish contribution to WHO-initiated survey Monitoring of Trends and Determinants in Cardiovascular Diseases [8–10], which was approved by the ethics committee for the Copenhagen Country and is following the Helsinki II declaration on human rights. All subjects in the study signed a letter of informed consent. Initially, 4807 Danish citizens, aged 30, 40, 50 or 60 years living in the western part of Copenhagen County were invited onto the study and 3608 attended the health examination and were of Danish origin [10–12].

### Dietary variables

All subjects were invited to complete a diet diary. Finally, 1852 subjects (903 men and 949 women) completed 7-day weighed diet diary at baseline. Subjects with previous Cardiometabolic Disease (*n* = 73) or fracture (*n* = 33) were excluded from the baseline, giving a total of 1746 subjects for the current analysis.

The 7-day weighted diet diaries were completed using a pre-coded form containing 111 foods and foods groups, in cooperation with a guide on the size of standard measure if weighing the food was not possible [8, 11]. Dairy products were recoded as milk (skimmed, partly skimmed, whole, buttermilk, buttermilk with lemon, milk with chocolate); soured milk products; yogurt with fruits; cream (13% fat, whipping 38% fat, cultured 18% fat); cheese (all varieties of fresh unripened cheese, unripened smoked cheese, and Danish blue cheese) and butter (salt added). Total dairy was calculated by summing up different type of dairy products. Total fermented dairy was calculated by summing up buttermilk, cheese, yogurt with fruits, and soured milk products. Low-fat milk was calculated by summing up skimmed and partly skimmed milk.7-day weighted diet diaries was compared with food-frequency questionnaire, which showed a fairly high degree of correlation (milk and yogurt: 0.66 (women) and 0.68 (men); cheese: 0.50 (women) and 0.56 (men); butter, lard and hard margarine: 0.42 (men) and 0.44 (women) [11].

### Incidence of CVD, CHD, fracture, hypertension and all-cause mortality

Total outcome events of CVD, CHD, and fracture (including fatal and non-fatal events) were defined according to the International Classification of Diseases. Diagnosis codes for CVD, CHD and fracture were ICD-8 and ICD-10. Cases were identified by recorded linkage to the National Patient Registry [13–15]. Blood pressure was measured by a trained nurse with hypertension defined as blood pressure above 140/90 mm Hg or taking antihypertensive medication [10, 16]. Furthermore, the records of all subjects in the National Patient Registry were flagged so that notification of death certificate was received automatically. Documentation of Death Registry has been published earlier [13].

### Other measurements

Information on age, gender, family history of myocardial infarction, education, smoking habits, leisure-time physical activity, live alone and multivitamin supplement use was obtained by a self-administered questionnaire. Body mass index (BMI) was calculated as weight (kg)/ height (m^2^). Systolic and diastolic blood pressure were measured after at least 5 mins rest. Also, serum total cholesterol and triacylglycerols were measured in fasting serum samples at baseline.

### Statistical analysis

All data analysis was conducted using STATA (version 16.1; STATA Corporation, 2020) and a 2-sided *P*<0.05 was considered statistically significant. In the longitudinal analysis, Cox proportional hazard models with age as the underlying time variable were used to calculate non-adjusted and multivariable adjusted hazard ratios (HR) by comparing the time until onset of disease or mortality in subjects in the highest intake categories of dairy consumption with that in the lowest dairy group as the reference group. The survival time for each endpoint (CVD, CHD, fracture, and all-cause mortality) was the first occurrence of an endpoint, or death, or the date of receipt of the last follow-up questionnaire (the loss of the follow-up was considered as one type of completeness). If subjects had a non-fatal event these data were used rather than the later date of death. The end of follow-up was September 2012. In total, subjects were followed for almost 30 years.

The first multivariable model controlled for several confounding factors. These included the covariates gender, BMI (kg/m^2^), food energy intake (kJ/day), alcohol consumption (g/day, non-drinkers, drinkers by tertile), education (7 years or less, 8–11 years, 12 years or more), smoking (never smoker, current smoker, former smoker), physical activity (sedentary, moderate, active), family history of myocardial infarction (MI) (yes or no), multivitamin (yes or no). The second multivariable model also controlled for serum total cholesterol (mmol/L), serum triaclyglycerols (mmol/L) and incidence of hypertension (yes or no).

The possibility of an interaction between dairy consumption and gender with respect to any of the outcomes was investigated by an analysis including an interaction term in the Cox proportional hazard models. Baseline characteristics were assessed by linear regression or logistic regression for continuous variables and categorical variable, respectively.

## Results

### Baseline characteristics according to dairy consumption

The mean weekly total dairy consumption of 1746 subjects were 365.1 (SD = 244.4) grams and ranged from 0 to 2086.1 grams per week. The baseline characteristics of the subjects are shown in Table 1. The mean age was 47 years (SD = 11.0) and mean value of BMI was 24.5 kg/m^2^ (SD = 3.7), 52.2% subjects were women. The subjects in the highest quartiles of total dairy consumption were significantly more likely to be younger, male, non-smokers, lower BMI, higher food energy intake, active physical activity, higher education (at least 12 years), more multivitamin supplement users, and consume less alcohol. They also had a lower incidence of hypertension, family history of MI, serum cholesterol, triacylglycerols, and blood pressure (systolic blood pressure and diastolic blood pressure). After controlling for total energy from foods, the subjects with the highest total dairy consumption had a significantly higher intake of protein and carbohydrate but not fat.

**Table 1:** Baseline characteristics of participants according to quartiles of weekly dairy consumption^1^.

Weekly total dairy consumption (n, g/wk)					
Variables	0≤n<192	192≤n<315	315≤n<480	480≤n	*P* for trend^2^
No. of subjects	437	436	437	436	
Mean intake (SD), g	119.6 (48.1)	253.3 (36.4)	391.1 (46.3)	697.0 (223.1)	-
Age, y	48.8 (10.6)	48.3 (10.5)	47.2 (11.3)	43.9 (10.8)	<0.001
Female, %	55	54	58	43	0.003
Body mass index, mean (SD) kg/m^2^	24.9 (3.6)	24.5 (3.7)	24.0 (3.5)	24.4 (3.8)	0.004
Smoking, %					0.001
Never smoker	19	26	30	30	
Current smoker	58	54	50	51	
Formal smoker	23	20	20	19	
Physical activity, %					0.004
Sedentary	29	22	25	22	
Moderate	53	59	54	51	
Active	18	19	21	27	
Education, %					<0.001
0–7 yrs	45	40	37	31	
8–11 yrs	49	54	52	55	
At least 12 yrs	6	6	11	14	
Live alone, %	9.8	9.9	10.3	13.3	0.10
Energy intake, mean (SD) kJ/d	7870 (2583)	8827 (2689)	9186 (2566)	11182 (3330)	<0.001
Fat, % of total energy	43.8	44.5	44.1	43.1	0.06
Protein, % of total energy	13.9	14.2	14.4	15.0	<0.001
Carbohydrate, % of total energy	34.7	35.5	36.7	37.8	<0.001
Alcohol intake, mean (SD) g/d	21.3 (23.9)	18.2 (20.3)	15.5 (16.8)	16.8 (20.3)	0.003
Multivitamin supplement, %	49.0	57.0	59.7	62.6	<0.001
Incidence of hypertension, %	19.7	13.8	13.0	13.8	0.016
Family history of MI, %	25.2	23.6	23.3	18.2	0.03
Serum total cholesterol, mean (SD) mmol/L	6.5 (1.3)	6.2 (1.1)	6.2 (1.2)	5.9 (1.2)	<0.001
Serum triacylglycerols, mean (SD) mmol/L	1.4 (1.1)	1.3 (0.7)	1.3 (1.1)	1.2 (0.6)	<0.001
Systolic blood pressure, mean (SD) mmHg	126.3 (17.7)	124.8 (17.2)	124.2 (17.0)	122.1 (15.6)	<0.001
Diastolic blood pressure, mean (SD) mmHg	78.5 (11.0)	77.2 (11.0)	77.0 (11.0)	76.7 (10.7)	0.017

^1^ All values are mean ± SD.

^2^ *P*-trend was assessed by linear regression (continuous variables) or by logistic regression (categorical variables).

### Dairy consumption and incidence of CVD, CHD, fracture, and all-cause mortality

During the follow-up of 30 years, incident cases of total CVD events (*n* = 904), CHD events (*n* = 332), fracture events (*n* = 447) and all-cause mortality (*n* = 680) were reported in the subjects initially free from CVD, T2D and fracture. Total dairy consumption was not associated with the incidence of CVD, CHD, fracture, or all-cause mortality (Table 2). When different types of dairy consumption were investigated separately for CVD, CHD, fracture, or all-cause mortality events, a significant trend of lower risk of fracture with increasing total fermented dairy consumption (adjusted model *P* = 0.02) was observed, with HR of 0.67 (95% CI: 0.50–0.90) for the highest (total fermented dairy>133 g/week) vs lowest (0≤total fermented dairy≤26, g/week) quartiles of total fermented dairy consumption (Table 3). There were no associations between total fermented dairy consumption and CVD, CHD, or all-cause mortality events. Furthermore, significant trends of lower risk of CVD, CHD and all-cause mortality with increasing weekly low-fat milk consumption (Table 4) were found: the HRs of the highest (low-fat milk>194 gram/week) vs lowest (0≤low-fat milk ≤19) are 0.84 (95% CI: 0.68–1.03), 0.82 (0.59–1.16) and 0.77 (0.61–0.97) for the CVD, CHD and all-cause mortality, respectively. There were no associations between total milk (S1 Table), full-fat milk (S2 Table), yogurt (S3 Table), cheese (S4 Table), butter (S5 Table) or cream (S6 Table) and CVD, CHD, fracture, or all-cause mortality events.

**Table 2:** Longitudinal study of incidence of CVD, CHD, fracture, and all-cause mortality according to quartiles of weekly total dairy consumption of all subjects.

	Total dairy consumption (n, g/wk)				
Characteristics	0≤n<192	192≤n<315	315≤n<480	480≤n	*P*-trend
Total subjects, n	437	436	437	436	
Mean intake (SD), g	119.6 (48.1)	253.3 (36.4)	391.1 (46.3)	697.0 (223.1)	
**Total CVD events**					
No. of events	239	239	226	200	
HR (non-adjust)	1	0.96 (0.80–1.15)	0.88 (0.74–1.06)	0.74 (0.61–0.89)	0.001
HR (adjusted Model 1)^1^	1	1.05 (0.86–1.28)	1.11 (0.90–1.36)	1.04 (0.83–1.31)	0.61
HR (adjusted Model 2)^2^	1	1.12 (0.92–1.37)	1.13 (0.95–1.43)	1.13 (0.90–1.43)	0.24
**Total CHD events**					
No. of events	105	74	88	65	
HR (non-adjust)	1	0.66 (0.49–0.89)	0.81 (0.61–1.07)	0.55 (0.41–0.75)	0.001
HR (adjusted Model 1)^1^	1	0.73 (0.53–1.02)	1.01 (0.73–1.39)	0.65 (0.45–0.95)	0.13
HR (adjusted Model 2)^2^	1	0.80 (0.57–1.11)	1.09 (0.79–1.51)	0.76 (0.52–1.11)	0.48
**Total fracture events**					
No. of events	120	114	106	107	
HR (non-adjust)	1	0.93 (0.72–1.20)	0.85 (0.66–1.11)	0.83 (0.64–1.07)	0.12
HR (adjusted Model 1)^1^	1	0.92 (0.70–1.22)	0.95 (0.71–1.26)	1.06 (0.77–1.45)	0.73
HR (adjusted Model 2)^2^	1	0.94 (0.71–1.24)	0.97 (0.73–1.29)	1.07 (0.78–1.48)	0.66
**All-cause mortality**					
No. of events	203	182	161	134	
HR (non-adjust)	1	0.84 (0.69–1.03)	0.71 (0.57–0.87)	0.57 (0.46–0.71)	<0.001
HR (adjusted Model 1)^1^	1	0.96 (0.77–1.19)	0.93(0.74–1.18)	0.75 (0.58–0.98)	0.05
HR (adjusted Model 2)^2^	1	0.99 (0.79–1.23)	0.98 (0.78–1.24)	0.85 (0.64–1.11)	0.28

^1^ Values are hazard ratios (95% CIs) derived by Cox proportional hazards regression models adjusted for gender, BMI, food energy intake, alcohol consumption, education, smoking, physical activity, family history of MI, multivitamin.

^2^ Adjusted as model 1 plus serum cholesterol, triglycerides, incidence of hypertension.

**Table 3:** Longitudinal study of incidence of CVD, CHD, fracture, and all-cause mortality according to quartiles of weekly total fermented dairy consumption of all subjects.

	Total fermented dairy (n, g/wk)				
Characteristics	0≤n≤26	26<n≤59	59<n≤133	133<n	*P*-trend
Total subjects, n	445	431	435	435	
Mean intake (SD), g	12.6 (9.0)	41.1 (9.1)	90.4 (22.2)	249.7 (137.3)	
**Total CVD events**					
No. of events	239	217	230	218	
HR (non-adjust)	1	0.90 (0.75–1.08)	0.94 (0.79–1.13)	0.88 (0.73–1.05)	0.24
HR (adjusted Model 1)^1^	1	0.92 (0.76–1.13)	1.05 (0.86–1.27)	1.06 (0.86–1.30)	0.37
HR (adjusted Model 2)^2^	1	0.96 (0.79–1.18)	1.11 (0.91–1.35)	1.15 (0.94–1.41)	0.10
**Total CHD events**					
No. of events	99	87	75	71	
HR (non-adjust)	1	0.89 (0.66–1.18)	0.74 (0.55–1.00)	0.70 (0.52–0.95)	0.01
HR (adjusted Model 1)^1^	1	0.92 (0.67–1.25)	0.77 (0.55–1.07)	0.94 (0.67–1.31)	0.45
HR (adjusted Model 2)^2^	1	0.96 (0.70–1.31)	0.85 (0.61–1.20)	1.05 (0.75–1.47)	0.99
**Total fracture events**					
No. of events	121	110	121	95	
HR (non-adjust)	1	0.92 (0.71–1.19)	1.00 (0.78–1.29)	0.76 (0.58–1.00)	0.10
HR (adjusted Model 1)^1^	1	0.91 (0.69–1.20)	1.00 (0.76–1.32)	0.71 (0.53–0.95)	0.05
HR (adjusted Model 2)^2^	1	0.90 (0.68–1.19)	1.00 (0.76–1.31)	0.67 (0.50–0.90)	0.02
**All-cause mortality**					
No. of events	198	174	160	148	
HR (non-adjust)	1	0.86 (0.70–1.05)	0.75 (0.61–0.93)	0.69 (0.56–0.86)	<0.001
HR (adjusted Model 1)^1^	1	0.89 (0.71–1.11)	0.78 (0.62–0.98)	0.91 (0.72–1.15)	0.23
HR (adjusted Model 2)^2^	1	0.94 (0.75–1.17)	0.86 (0.68–1.08)	0.99 (0.78–1.26)	0.70

^1^ Values are hazard ratios (95% CIs) derived by Cox proportional hazards regression models adjusted for gender, BMI, food energy intake, alcohol consumption, education, smoking, physical activity, family history of MI, multivitamin.

^2^ Adjusted as model 1 plus serum cholesterol, triglycerides, incidence of hypertension.

**Table 4:** Longitudinal study of incidence of CVD, CHD, fracture, and all-cause mortality according to quartiles of weekly low-fat milk consumption of all subjects^1^.

	Low-fat milk (n, g/wk)				
Characteristics	0≤n≤19	20<n≤58	58<n≤194	194<n	*P*-trend
Total subjects, n	437	436	437	436	
Mean intake (SD), g	8.1 (5.7)	36.4 (10.9)	110.3 (39.8)	400.4 (201.5)	
**Total CVD events**					
No. of events	260	237	209	196	
HR (non-adjust)	1	0.87 (0.73–1.03)	0.70 (0.59–0.84)	0.65 (0.54–0.78)	<0.001
HR (adjusted Model 1)^1^	1	0.87 (0.72–1.05)	0.77 (0.63–0.93)	0.80 (0.66–0.98)	0.012
HR (adjusted Model 2)^2^	1	0.87 (0.72–1.05)	0.76 (0.62–0.93)	0.84 (0.68–1.03)	0.03
**Total CHD events**					
No. of events	104	94	65	69	
HR (non-adjust)	1	0.88 (0.66–1.16)	0.57 (0.42–0.78)	0.60 (0.44–0.81)	<0.001
HR (adjusted Model 1)^1^	1	0.87 (0.64–1.18)	0.58 (0.42–0.82)	0.72 (0.51–1.01)	0.008
HR (adjusted Model 2)^2^	1	0.88 (0.65–1.20)	0.57 (0.40–0.80)	0.82 (0.59–1.16)	0.04
**Total fracture events**					
No. of events	110	122	110	105	
HR (non-adjust)	1	1.12 (0.87–1.45)	0.96 (0.74–1.25)	0.89 (0.68–1.16)	0.24
HR (adjusted Model 1)^1^	1	1.02 (0.78–1.35)	0.91 (0.69–1.21)	1.01 (0.76–1.34)	0.83
HR (adjusted Model 2)^2^	1	1.03 (0.78–1.35)	0.92 (0.69–1.23)	1.02 (0.77–1.37)	0.93
**All-cause mortality**					
No. of events	210	184	153	133	
HR (non-adjust)	1	0.82 (0.68–1.00)	0.65 (0.53–0.80)	0.54 (0.44–0.68)	<0.001
HR (adjusted Model 1)^1^	1	0.83 (0.67–1.02)	0.68 (0.54–0.86)	0.70 (0.55–0.88)	0.001
HR (adjusted Model 2)^2^	1	0.83 (0.67–1.03)	0.66 (0.53–0.83)	0.77 (0.61–0.97)	0.004

^1^ Values are hazard ratios (95% CIs) derived by Cox proportional hazards regression models adjusted for gender, BMI, food energy intake, alcohol consumption, education, smoking, physical activity, family history of MI, multivitamin.

^2^ Adjusted as model 1 plus serum cholesterol, triglycerides, incidence of hypertension.

In stratified analyses for gender, there was no consistent effect modification by gender. The P-values for effect modification by gender were significant for CVD events and fermented dairy consumption (P = 0.03), CHD events and total milk consumption (P = 0.01), and incidence of fracture and butter consumption (P = 0.01). When the analysis was performed in men and women separately, significant associations were found for increased risk of total CVD events with increasing total fermented dairy consumption in men (*P* = 0.007, S7 Table); decreased risk of total CHD events with increasing total milk consumption (*P* = 0.01, S7 Table) in women; and lower incidence of fracture with increasing butter consumption in men (*P* = 0.03, S7 Table). There was no effect modification by gender for other dairy categories for CVD, CHD, fracture events or all-cause mortality (data was not shown).

## Discussion

In the current population-based prospective cohort study of Danish citizens, high fermented dairy consumption was found to be associated with lower fracture risk, and also high intake of low-fat milk was associated with lower CVD, CHD and all-cause mortality risk. The total dairy and other dairy groups, including total milk, full-fat milk, yogurt, cheese, butter and cream, were not associated with risk of CVD, CHD, fracture and all-cause mortality.

We found a suggestive inverse association for fermented dairy intake and fracture risk. This is supported by the evidence of one recent meta-analysis of Bian *et al*. [6], which found a 25% lower risk of hip fracture (RR 0.75, 95% CI 0.66–0.86) associated with higher yogurt intake, and a 32% lower risk of hip fracture (RR 0.68, 95% CI 0.61–0.77) associated with higher cheese consumption by pooling data from the previous studies. There were no heterogeneities (*I*^*2*^ = 0%) for those two analyses. However, separating analyses of yogurt and cheese from total fermented dairy did not show the inverse associations with fracture risk in the current study. One possible reason may be related to the different types of cheese and yogurt included across different cohort studies. In the current study, the yogurt was defined as ‘yogurt with fruit’, and cheese includes all varieties of fresh unripened cheese, unripened smoked cheese, and Danish blue cheese. However, the definitions of yogurt and cheese in those included studies [7, 17, 18] in the meta-analysis [6] were not clear. Furthermore, the range of intakes may influence the associations. However, the range of intakes was not clearly reported in the study of Michaëlsson *et al*. [7], which reported the inverse associations of cheese or yogurt with fracture risk. Another possible reason may be related to the sample size. Separating yogurt and cheese from the total fermented dairy resulted in less statistical power in the current small cohort. Therefore, it was less likely to see a significant association.

Dairy fat is rich in saturated fat and has been hypothesised to have detrimental effects on CVD, thus, low-fat dairy has been recommended by several dietary guidelines [19–21]. However, emerging evidence of both observational studies and randomised controlled trials show that full-fat dairy products may have a neutral or even moderately beneficial effect on cardiometabolic health [3, 4, 22–24]. The findings of our study agree with the previous research that there were no negative associations between high-fat milk intake and CVD risk, but the recent meta-analysis [25] which reported each additional dairy 200 g of was positively associated with CHD (RR 1.08, 95% CI 1.00–1.16) by summarised four studies. However, heterogeneity was observed [25].

Very few prospective cohort studies have evaluated the relationship between low-fat milk and CVD or mortality risk [26–28]. We found inverse associations between low-fat milk intake and CVD, CHD and all-cause mortality risk, which is in line with the study of Sonestedt *et al*. [27] which showed that individuals with high compared to low consumption of low-fat milk tended to have a lower risk of CVD (HR 0.88, 95%CI: 0.76–1.01), although the association was not statistically significant (*P* = 0.08). However, the other two studies [26, 28] did not find the associations between low-fat milk and CVD or mortality risk. One possible reason may be related to the follow-up time. The current studies have a longer follow-up of 30 years than the two other studies of 10–11.6 years [26, 28]. Therefore, more evidence of prospective cohort studies and RCT is needed to verify the current findings of whether low-fat milk is associated with lower CVD morbidity and mortality risks.

In stratified analyses, the intake of fermented dairy was associated with higher CVD risk in men, whereas intake of milk and butter was associated with lower CHD and fracture risk in women. These findings imply that the relationship between dairy consumption and disease outcomes may be dependent on sex, which was also reported by previous cohort studies [7, 29]. However, our results are not in line with the cohort study of Michaëlsson *et al*. [7], with suggested high milk intake was associated with higher fracture risk in Swedish women but not in Swedish men. One possible reason for the different results across different cohorts may be related to the various lifestyle of the study subjects. In the current study, subjects with higher dairy consumption tended to have a healthier lifestyle (e.g. higher education rate, less smoker, more vitamin supplement users), whereas, subjects in the study of Michaëlsson *et al*. [7] who had a higher milk consumption tended to have a relatively unhealthier lifestyle (e.g. lower education rate, more smokers, less vitamin users).

Underlying mechanisms remain unknown for the beneficial effect of the fermented dairy consumption on health outcome. Previous studies suggested the potential mechanism may be linked to dairy components could improve glucose tolerance and promote gut microbial population shifts [23, 30]. Future studies to elucidate potential mechanisms of the beneficial effect of fermented dairy and possible low-fat milk intake on cardiometabolic health are important and needed.

The present study with the prospective design has its strengths and weaknesses. A major strength of the MONICA study is that it has a follow-up over 30 years, which is one of the longest worldwide prospective cohort studies monitoring the dietary risk factors for CVD and fracture events. Another strength is that food intake was recorded in 7-day weighed diet diary, which provides detailed weight intake for different types of dairy groups. Furthermore, identification of diseases and mortality was obtained via linkage to the National Patient Registry, which provides accurate event ascertainment. Last but not least, this representative Danish population-based cohort with prospective study design has advantages of reducing the selection bias [31]. However, the current findings should be seen in light of some limitations. A limitation is dairy consumption was only recorded at baseline not during the following-up, which may have affected the observed associations. Another limitation is that prospective cohort studies cannot provide evidence of the causality effect for dairy consumption and disease outcomes or mortality. However, no RCTs with dairy products as the main intervention and sufficiently long follow-up for cardiovascular endpoints have been conducted [3] and likely may never be conducted. Previous meta-analyses [32–35] summarised the available evidence from RCTs in relation to cardiovascular risk factors, and concluded that dairy or dairy components are unlikely to have detrimental effects on the cardiometabolic health, and fermented dairy consumption (e.g. yogurt and cheese) have potential roles in protecting against cardiometabolic disease. Furthermore, although we adjusted for a range of potential confounders, residual confounding factors might still have influenced the associations.

## Conclusions

In conclusion, the present study found that high intake of total fermented dairy was related to a lower fracture risk, and also that a high intake of low-fat milk was related to a lower risk of incident CVD and CHD and of all-cause mortality. The mechanisms linking fermented dairy and low-fat milk consumption to the health outcomes are not clear. Healthy lifestyle or the specific foods dairy products replace in the general diet may contribute to the detected associations and this needs future research to confirm.

## Supporting information

S1 Table Longitudinal study of incidence of CVD, CHD, fracture, and all-cause mortality according to quartiles of weekly total milk consumption of all subjects. (DOCX)

S2 Table Longitudinal study of incidence of CVD, CHD, fracture, and all-cause mortality according to quartiles of weekly full-fat milk consumption of all subjects. (DOCX)

S3 Table Longitudinal study of incidence of CVD, CHD, fracture, and all-cause mortality according to quartiles of weekly yogurt consumption of all subjects. (DOCX)

S4 Table Longitudinal study of incidence of CVD, CHD, fracture, and all-cause mortality according to quartiles of weekly cheese consumption of all subjects. (DOCX)

S5 Table Longitudinal study of incidence of CVD, CHD, fracture, and all-cause mortality according to quartiles of weekly butter consumption of all subjects. (DOCX)

S6 Table Longitudinal study of incidence of CVD, CHD, fracture, and all-cause mortality according to quartiles of weekly cream consumption of all subjects. (DOCX)

S7 Table Multivariate RR (95% CI)^1^ of incidence of a) CVD and total dairy; b) CVD and total fermented dairy; c) fracture and butter consumption, stratified by gender. (DOCX)
