# Supplementary material for: Association between dairy consumption and cardiovascular disease events, bone fracture and all-cause mortality
Source: PLoS One. 2022 Sep 9;17(9):e0271168. doi: 10.1371/journal.pone.0271168 (PMC9462570; doi:10.1371/journal.pone.0271168)
Supplement: S1 Table — (DOCX) [file pone.0271168.s001.docx]

**S1 Table.** Longitudinal study of incidence of CVD, CHD, fracture, and all-cause mortality according to quartiles of weekly total milk consumption of all subjects^1^.

|  | Total milk (n, g/wk) | | | |  |
| --- | --- | --- | --- | --- | --- |
| Characteristics | 0≤n≤65 | 65<n≤173 | 173<n≤320 | 320<n | *P*-trend |
| Total subjects, n | 437 | 436 | 437 | 436 |  |
| Mean intake (SD), g | 26.4 (19.1) | 116.9 (32.3) | 237.3 (42.7) | 533.4 (210.8) |  |
| **Total CVD events** |  |  |  |  |  |
| No. of events | 255 | 230 | 212 | 207 |  |
| HR (non-adjust) | 1 | 0.84 (0.70-1.01) | 0.74 (0.62-0.89) | 0.72 (0.60-0.86) | <0.001 |
| HR (adjusted Model 1)^1^ | 1 | 0.86 (0.71-1.04) | 0.86 (0.70-1.05) | 0.90 (0.73-1.12) | 0.35 |
| HR (adjusted Model 2)^2^ | 1 | 0.82 (0.67-0.99) | 0.86 (0.70-1.05) | 0.91 (0.73-1.12) | 0.44 |
| **Total CHD events** |  |  |  |  |  |
| No. of events | 101 | 81 | 81 | 69 |  |
| HR (non-adjust) | 1 | 0.76 (0.57-1.02) | 0.76 (0.57-1.01) | 0.62 (0.45-0.84) | 0.003 |
| HR (adjusted Model 1)^1^ | 1 | 0.80 (0.58-1.11) | 0.84 (0.61-1.17) | 0.68 (0.48-0.98) | 0.06 |
| HR (adjusted Model 2)^2^ | 1 | 0.74 (0.54-1.02) | 0.85 (0.61-1.19) | 0.72 (0.50-1.03) | 0.14 |
| **Total fracture events** |  |  |  |  |  |
| No. of events | 118 | 125 | 98 | 106 |  |
| HR (non-adjust) | 1 | 1.06 (0.82-1.36) | 0.78 (0.60-1.02) | 0.84 (0.64-1.09) | 0.05 |
| HR (adjusted Model 1)^1^ | 1 | 1.09 (0.83-1.42) | 0.85 (0.64-1.14) | 1.08 (0.80-1.45) | 0.92 |
| HR (adjusted Model 2)^2^ | 1 | 1.10 (0.83-1.44) | 0.87 (0.65-1.17) | 1.10 (0.81-1.49) | 0.95 |
| **All-cause mortality** |  |  |  |  |  |
| No. of events | 194 | 186 | 154 | 146 |  |
| HR (non-adjust) | 1 | 0.92 (0.76-1.13) | 0.72 (0.58-0.89) | 0.68 (0.55-0.84) | <0.001 |
| HR (adjusted Model 1)^1^ | 1 | 0.98 (0.79-1.22) | 0.82 (0.65-1.04) | 0.84 (0.66-1.08) | 0.08 |
| HR (adjusted Model 2)^2^ | 1 | 0.92 (0.74-1.15) | 0.82 (0.65-1.04) | 0.88 (0.68-1.13) | 0.19 |

^1^ Values are hazard ratios (95 % CIs) derived by Cox proportional hazards regression models adjusted for gender, BMI, food energy intake, alcohol consumption, education, smoking, physical activity, family history of MI, multivitamin.

^2^ Adjusted as model 1 plus serum cholesterol, triglycerides, incidence of hypertension.
