# Supplementary material for: Association between dairy consumption and cardiovascular disease events, bone fracture and all-cause mortality
Source: PLoS One. 2022 Sep 9;17(9):e0271168. doi: 10.1371/journal.pone.0271168 (PMC9462570; doi:10.1371/journal.pone.0271168)
Supplement: S2 Table — (DOCX) [file pone.0271168.s002.docx]

**S2 Table.** Longitudinal study of incidence of CVD, CHD, fracture, and all-cause mortality according to quartiles of weekly full-fat milk consumption of all subjects^1^.

|  | Full-fat milk (n, g/wk) | | | |  |
| --- | --- | --- | --- | --- | --- |
| Characteristics | None | 3≤n≤86 | 86<n≤182 | 182<n | *P*-trend |
| Total subjects, n | 914 | 305 | 251 | 276 |  |
| Mean intake (SD), g | 0 | 45.6 (23.4) | 134.4 (25.8) | 349.3 (188.2) |  |
| **Total CVD events** |  |  |  |  |  |
| No. of events | 462 | 162 | 140 | 140 |  |
| HR (non-adjust) | 1 | 1.04 (0.87-1.25) | 1.11 (0.92-1.34) | 0.99 (0.82-1.19) | 0.76 |
| HR (adjusted Model 1)^1^ | 1 | 1.01 (0.83-1.22) | 1.09 (0.59-1.33) | 1.02 (0.83-1.26) | 0.92 |
| HR (adjusted Model 2)^2^ | 1 | 0.99 (0.81-1.20) | 1.05 (0.86-1.29) | 0.98 (0.79-1.21) | 0.96 |
| **Total CHD events** |  |  |  |  |  |
| No. of events | 166 | 62 | 53 | 51 |  |
| HR (non-adjust) | 1 | 1.15 (0.86-1.83) | 1.18 (0.87-1.61) | 1.02 (0.75-1.40) | 0.57 |
| HR (adjusted Model 1)^1^ | 1 | 1.25 (0.91-1.72) | 1.20 (0.86-1.67) | 0.99 (0.69-1.41) | 0.64 |
| HR (adjusted Model 2)^2^ | 1 | 1.19 (0.86-1.63) | 1.15 (0.83-1.61) | 0.93 (0.65-1.32) | 0.97 |
| **Total fracture events** |  |  |  |  |  |
| No. of events | 243 | 88 | 59 | 57 |  |
| HR (non-adjust) | 1 | 1.11 (0.87-1.42) | 0.89 (0.67-1.18) | 0.75 (0.56-1.00) | 0.05 |
| HR (adjusted Model 1)^1^ | 1 | 1.07 (0.82-1.38) | 0.87 (0.64-1.17) | 0.86 (0.63-1.17) | 0.26 |
| HR (adjusted Model 2)^2^ | 1 | 1.05 (0.81-1.37) | 0.87 (0.64-1.18) | 0.88 (0.64-1.18) | 0.28 |
| **All-cause mortality** |  |  |  |  |  |
| No. of events | 342 | 121 | 107 | 110 |  |
| HR (non-adjust) | 1 | 1.07 (0.87-1.31) | 1.14 (0.92-1.42) | 1.09 (0.88-1.35) | 0.27 |
| HR (adjusted Model 1)^1^ | 1 | 1.05 (0.84-1.31) | 1.06 (0.84-1.34) | 1.11 (0.87-1.41) | 0.37 |
| HR (adjusted Model 2)^2^ | 1 | 1.01 (0.81-1.27) | 1.02 (0.81-1.29) | 1.08 (0.85-1.37) | 0.58 |

^1^ Values are hazard ratios (95 % CIs) derived by Cox proportional hazards regression models adjusted for gender, BMI, food energy intake, alcohol consumption, education, smoking, physical activity, family history of MI, multivitamin.

^2^ Adjusted as model 1 plus serum cholesterol, triglycerides, incidence of hypertension.
