# Supplementary material for: Association between dairy consumption and cardiovascular disease events, bone fracture and all-cause mortality
Source: PLoS One. 2022 Sep 9;17(9):e0271168. doi: 10.1371/journal.pone.0271168 (PMC9462570; doi:10.1371/journal.pone.0271168)
Supplement: S3 Table — (DOCX) [file pone.0271168.s003.docx]

**S3 Table.** Longitudinal study of incidence of CVD, CHD, fracture, and all-cause mortality according to quartiles of weekly yogurt consumption of all subjects^1^.

|  | Yogurt (n, g/wk) | | | |  |
| --- | --- | --- | --- | --- | --- |
| Characteristics | None | 0<n≤50 | 50<n≤120 | 120<n | *P*-trend |
| Total subjects, n | 1322 | 145 | 145 | 134 |  |
| Mean intake (SD), g | 0 | 28.0 (12.0) | 81.3 (19.3) | 194.7 (81.7) |  |
| **Total CVD events** |  |  |  |  |  |
| No. of events | 690 | 71 | 74 | 69 |  |
| HR (non-adjust) | 1 | 0.89 (0.69-1.13) | 0.96 (0.76-1.22) | 0.96 (0.75-1.23) | 0.57 |
| HR (adjusted Model 1)^1^ | 1 | 1.04 (0.81-1.36) | 1.20 (0.93-1.54) | 1.11 (0.93-1.54) | 0.36 |
| HR (adjusted Model 2)^2^ | 1 | 1.02 (0.79-1.33) | 1.17 (0.90-1.50) | 1.13 (0.87-1.48) | 0.19 |
| **Total CHD events** |  |  |  |  |  |
| No. of events | 259 | 25 | 28 | 20 |  |
| HR (non-adjust) | 1 | 0.85 (0.56-1.28) | 0.98 (0.66-1.45) | 0.73 (0.46-1.15) | 0.21 |
| HR (adjusted Model 1)^1^ | 1 | 1.16 (0.74-1.80) | 1.40 (0.92-2.12) | 0.88 (0.54-1.45) | 0.65 |
| HR (adjusted Model 2)^2^ | 1 | 1.20 (0.77-1.87) | 1.46 (0.96-2.21) | 0.95 (0.58-1.56) | 0.42 |
| **Total fracture events** |  |  |  |  |  |
| No. of events | 342 | 38 | 39 | 28 |  |
| HR (non-adjust) | 1 | 0.98 (0.70-1.36) | 1.03 (0.74-1.43) | 0.77 (0.53-1.14) | 0.33 |
| HR (adjusted Model 1)^1^ | 1 | 0.81 (0.57-1.16) | 0.88 (0.62-1.26) | 0.73 (0.48-1.10) | 0.09 |
| HR (adjusted Model 2)^2^ | 1 | 0.82 (0.57-1.17) | 0.88 (0.61-1.26) | 0.73 (0.48-1.10) | 0.09 |
| **All-cause mortality** |  |  |  |  |  |
| No. of events | 541 | 43 | 52 | 44 |  |
| HR (non-adjust) | 1 | 0.64 (0.47-0.88) | 0.85 (0.64-1.13) | 0.77 (0.57-1.05) | 0.02 |
| HR (adjusted Model 1)^1^ | 1 | 0.79 (0.57-1.10) | 0.97 (0.71-1.33) | 1.01 (0.73-1.39) | 0.77 |
| HR (adjusted Model 2)^2^ | 1 | 0.82 (0.59-1.14) | 1.01 (0.74-1.39) | 1.05 (0.76-1.45) | 0.95 |

^1^ Values are hazard ratios (95 % CIs) derived by Cox proportional hazards regression models adjusted for gender, BMI, food energy intake, alcohol consumption, education, smoking, physical activity, family history of MI, multivitamin.

^2^ Adjusted as model 1 plus serum cholesterol, triglycerides, incidence of hypertension.
