# Supplementary material for: Association between dairy consumption and cardiovascular disease events, bone fracture and all-cause mortality
Source: PLoS One. 2022 Sep 9;17(9):e0271168. doi: 10.1371/journal.pone.0271168 (PMC9462570; doi:10.1371/journal.pone.0271168)
Supplement: S4 Table — (DOCX) [file pone.0271168.s004.docx]

**S4 Table.** Longitudinal study of incidence of CVD, CHD, fracture, and all-cause mortality according to quartiles of weekly cheese consumption of all subjects^1^.

|  | Cheese (n, g/wk) | | | |  |
| --- | --- | --- | --- | --- | --- |
| Characteristics | 0≤n≤15 | 15<n≤30 | 30<n≤49 | 49<n | *P*-trend |
| Total subjects, n | 441 | 458 | 415 | 432 |  |
| Mean intake (SD), g | 6.3 (5.2) | 23.3 (4.5) | 39.3 (5.1) | 73.7 (27.3) |  |
| **Total CVD events** |  |  |  |  |  |
| No. of events | 232 | 239 | 207 | 226 |  |
| HR (non-adjust) | 1 | 0.97 (0.81-1.16) | 0.93 (0.77-1.13) | 0.98 (0.82-1.18) | 0.76 |
| HR (adjusted Model 1)^1^ | 1 | 0.97 (0.81-1.18) | 0.98 (0.80-1.20) | 1.01 (0.82-1.24) | 0.96 |
| HR (adjusted Model 2)^2^ | 1 | 0.91 (0.75-1.10) | 0.95 (0.77-1.16) | 1.02 (0.83-1.25) | 0.80 |
| **Total CHD events** |  |  |  |  |  |
| No. of events | 90 | 82 | 80 | 80 |  |
| HR (non-adjust) | 1 | 0.87 (0.65-1.18) | 0.94 (0.70-1.27) | 0.89 (0.66-1.21) | 0.58 |
| HR (adjusted Model 1)^1^ | 1 | 0.85 (0.62-1.17) | 0.99 (0.71-1.39) | 0.89 (0.63-1.24) | 0.66 |
| HR (adjusted Model 2)^2^ | 1 | 0.86 (0.62-1.18) | 1.00 (0.72-1.40) | 0.92 (0.66-1.29) | 0.83 |
| **Total fracture events** |  |  |  |  |  |
| No. of events | 129 | 122 | 99 | 97 |  |
| HR (non-adjust) | 1 | 0.92 (0.72-1.18) | 0.79 (0.61-1.03) | 0.73 (0.56-0.95) | 0.010 |
| HR (adjusted Model 1)^1^ | 1 | 0.94 (0.72-1.23) | 0.86 (0.65-1.15) | 0.81 (0.60-1.08) | 0.12 |
| HR (adjusted Model 2)^2^ | 1 | 0.94 (0.72-1.22) | 0.85 (0.64-1.13) | 0.80 (0.60-1.08) | 0.11 |
| **All-cause mortality** |  |  |  |  |  |
| No. of events | 183 | 197 | 142 | 158 |  |
| HR (non-adjust) | 1 | 1.05 (0.86-1.28) | 0.77 (0.62-0.95) | 0.85 (0.68-1.05) | 0.018 |
| HR (adjusted Model 1)^1^ | 1 | 1.08 (0.87-1.34) | 0.88 (0.69-1.12) | 0.96 (0.75-1.22) | 0.41 |
| HR (adjusted Model 2)^2^ | 1 | 1.06 (0.85-1.31) | 0.89 (0.70-1.13) | 0.96 (0.76-1.22) | 0.46 |

^1^ Values are hazard ratios (95 % CIs) derived by Cox proportional hazards regression models adjusted for gender, BMI, food energy intake, alcohol consumption, education, smoking, physical activity, family history of MI, multivitamin.

^2^ Adjusted as model 1 plus serum cholesterol, triglycerides, incidence of hypertension.
