# Supplementary material for: Association between dairy consumption and cardiovascular disease events, bone fracture and all-cause mortality
Source: PLoS One. 2022 Sep 9;17(9):e0271168. doi: 10.1371/journal.pone.0271168 (PMC9462570; doi:10.1371/journal.pone.0271168)
Supplement: S5 Table — (DOCX) [file pone.0271168.s005.docx]

**S5 Table.** Longitudinal study of incidence of CVD, CHD, fracture, and all-cause mortality according to quartiles of weekly butter consumption of all subjects^1^.

|  | Butter (n, g/wk) | | | |  |
| --- | --- | --- | --- | --- | --- |
| Characteristics | 0≤n≤9.6 | 9.6<n≤20 | 20<n≤35 | 35<n | *P*-trend |
| Total subjects, n | 438 | 435 | 437 | 436 |  |
| Mean intake (SD), g | 4.3 (3.1) | 15.0 (3.2) | 27.6 (4.1) | 53.5 (18.9) |  |
| **Total CVD events** |  |  |  |  |  |
| No. of events | 243 | 217 | 220 | 224 |  |
| HR (non-adjust) | 1 | 0.85 (0.70-1.02) | 0.86 (0.72-1.04) | 0.90 (0.75-1.08) | 0.31 |
| HR (adjusted Model 1)^1^ | 1 | 0.95 (0.78-1.15) | 0.99 (0.78-1.15) | 1.02 (0.82-1.26) | 0.80 |
| HR (adjusted Model 2)^2^ | 1 | 0.96 (0.78-1.17) | 0.97 (0.80-1.19) | 0.96 (0.78-1.19) | 0.78 |
| **Total CHD events** |  |  |  |  |  |
| No. of events | 88 | 84 | 75 | 85 |  |
| HR (non-adjust) | 1 | 0.96 (0.71-1.29) | 0.84 (0.61-1.14) | 0.99 (0.73-1.33) | 0.71 |
| HR (adjusted Model 1)^1^ | 1 | 1.14 (0.82-1.58) | 0.94 (0.67-1.31) | 0.99 (0.70-1.41) | 0.69 |
| HR (adjusted Model 2)^2^ | 1 | 1.19 (0.86-1.65) | 0.93 (0.66-1.30) | 0.90 (0.64-1.29) | 0.34 |
| **Total fracture events** |  |  |  |  |  |
| No. of events | 123 | 111 | 107 | 106 |  |
| HR (non-adjust) | 1 | 0.88 (0.68-1.14) | 0.85 (0.66-1.11) | 0.87 (0.67-1.12) | 0.26 |
| HR (adjusted Model 1)^1^ | 1 | 0.87 (0.66-1.14) | 0.91 (0.69-1.21) | 1.00 (0.74-1.34) | 0.99 |
| HR (adjusted Model 2)^2^ | 1 | 0.86 (0.66-1.14) | 0.91 (0.69-1.20) | 0.99 (0.73-1.33) | 0.97 |
| **All-cause mortality** |  |  |  |  |  |
| No. of events | 172 | 169 | 160 | 179 |  |
| HR (non-adjust) | 1 | 0.97 (0.78-1.19) | 0.90 (0.73-1.12) | 1.05 (0.85-1.30) | 0.79 |
| HR (adjusted Model 1)^1^ | 1 | 1.08 (0.86-1.36) | 1.02 (0.80-1.30) | 1.15 (0.89-1.48) | 0.38 |
| HR (adjusted Model 2)^2^ | 1 | 1.12 (0.89-1.41) | 1.04 (0.82-1.31) | 1.09 (0.85-1.40) | 0.66 |

^1^ Values are hazard ratios (95 % CIs) derived by Cox proportional hazards regression models adjusted for gender, BMI, food energy intake, alcohol consumption, education, smoking, physical activity, family history of MI, multivitamin.

^2^ Adjusted as model 1 plus serum cholesterol, triglycerides, incidence of hypertension..
