# Supplementary material for: Association between dairy consumption and cardiovascular disease events, bone fracture and all-cause mortality
Source: PLoS One. 2022 Sep 9;17(9):e0271168. doi: 10.1371/journal.pone.0271168 (PMC9462570; doi:10.1371/journal.pone.0271168)
Supplement: S6 Table — (DOCX) [file pone.0271168.s006.docx]

**S6 Table.** Longitudinal study of incidence of CVD, CHD, fracture, and all-cause mortality according to quartiles of weekly cream consumption of all subjects^1^.

|  | Cream (n, g/wk) | | | |  |
| --- | --- | --- | --- | --- | --- |
| Characteristics | None | 0<n≤5.5 | 5.5<n<19 | 19≤n | *P*-trend |
| Total subjects, n | 535 | 347 | 428 | 436 |  |
| Mean intake (SD), g | 0 | 2.8 (1.5) | 11.5 (3.9) | 40.9 (25.9) |  |
| **Total CVD events** |  |  |  |  |  |
| No. of events | 306 | 167 | 213 | 218 |  |
| HR (non-adjust) | 1 | 0.76 (0.63-0.91) | 0.78 (0.66-0.93) | 0.80 (0.67-0.95) | 0.011 |
| HR (adjusted Model 1)^1^ | 1 | 0.85 (0.69-1.05) | 0.91 (0.75-1.10) | 0.96 (0.79-1.17) | 0.75 |
| HR (adjusted Model 2)^2^ | 1 | 0.87 (0.71-1.07) | 0.93 (0.76-1.12) | 0.95 (0.78-1.16) | 0.67 |
| **Total CHD events** |  |  |  |  |  |
| No. of events | 120 | 60 | 69 | 83 |  |
| HR (non-adjust) | 1 | 0.73 (0.53-0.99) | 0.67 (0.50-0.90) | 0.82 (0.62-1.08) | 0.08 |
| HR (adjusted Model 1)^1^ | 1 | 0.88 (0.62-1.23) | 0.81 90.59-1.13) | 1.10 (0.80-1.51) | 0.83 |
| HR (adjusted Model 2)^2^ | 1 | 0.89 (0.63-1.25) | 0.80 (0.57-1.11) | 1.05 (0.76-1.44) | 0.96 |
| **Total fracture events** |  |  |  |  |  |
| No. of events | 151 | 87 | 99 | 110 |  |
| HR (non-adjust) | 1 | 0.82 (0.63-1.07) | 0.77 (0.60-0.99) | 0.84 (0.66-1.08) | 0.11 |
| HR (adjusted Model 1)^1^ | 1 | 0.79 (0.60-1.05) | 0.75 (0.57-0.98) | 0.82 (0.62-1.08) | 0.12 |
| HR (adjusted Model 2)^2^ | 1 | 0.79 (0.59-1.05) | 0.74 (0.56-0.98) | 0.81 (0.61-1.07) | 0.11 |
| **All-cause mortality** |  |  |  |  |  |
| No. of events | 260 | 118 | 137 | 165 |  |
| HR (non-adjust) | 1 | 0.62 (0.50-0.77) | 0.56 (0.46-0.69) | 0.69 (0.57-0.84) | <0.001 |
| HR (adjusted Model 1)^1^ | 1 | 0.75 (0.59-0.96) | 0.74 (0.59-0.93) | 0.95 (0.76-1.18) | 0.69 |
| HR (adjusted Model 2)^2^ | 1 | 0.76 (0.60-0.97) | 0.74 (0.59-0.93) | 0.92 (0.73-1.15) | 0.26 |

^1^ Values are hazard ratios (95 % CIs) derived by Cox proportional hazards regression models adjusted for gender, BMI, food energy intake, alcohol consumption, education, smoking, physical activity, family history of MI, multivitamin.

^2^ Adjusted as model 1 plus serum cholesterol, triglycerides, incidence of hypertension.
