# Supplementary material for: Association between dairy consumption and cardiovascular disease events, bone fracture and all-cause mortality
Source: PLoS One. 2022 Sep 9;17(9):e0271168. doi: 10.1371/journal.pone.0271168 (PMC9462570; doi:10.1371/journal.pone.0271168)
Supplement: S7 Table — (DOCX) [file pone.0271168.s007.docx]

**S7 Table.** Multivariate RR (95% CI)^1^ of incidence of a) CVD and total dairy; b) CVD and total fermented dairy; c) fracture and butter consumption, stratified by gender.

| **a) Multivariate RR (95% CI) of incidence of CVD and total fermented dairy consumption, stratified by gender.** | | | | |  | |
| --- | --- | --- | --- | --- | --- | --- |
|  | Total fermented dairy (n, g/wk) | | | | | P-trend |
|  | 0≤n≤26 | 26<n≤59 | 59<n≤133 | 133<n | |  |
| Characteristics | **Men** | | | | |  |
| Subjects, n | 234 | 231 | 211 | 159 | |  |
| No. of events | 119 | 126 | 122 | 87 | |  |
| HR (95% CI) | 1 | 1.18 (0.90-1.55) | 1.30 (0.99-1.73) | 1.50 (1.11-2.02) | | 0.007 |
|  | **Women** | | | | |  |
| subjects, n | 211 | 200 | 224 | 276 | |  |
| No. of events | 120 | 91 | 108 | 131 | |  |
| HR (95% CI) | 1 | 0.73 (0.55-0.99) | 0.89 (0.67-1.19) | 0.83 (0.63-1.11) | | 0.48 |
| **b) Multivariate RR (95% CI) of incidence of CHD and total milk consumption, stratified by gender.** | | | |  | |  |
|  | Milk (n, g/wk) | | | | | P-trend |
|  | 0≤n≤65 | 65<n≤173 | 173<n≤320 | 320<n | |  |
| Characteristics | **Men** | | | | |  |
| Total subjects, n | 186 | 190 | 201 | 258 | |  |
| No. of events | 51 | 44 | 54 | 52 | |  |
| HR (95% CI) | 1 | 0.88 (0.55-1.39) | 1.30 (0.83-2.03) | 0.97 (0.61-1.55) | | 0.73 |
|  | **Women** | | | | |  |
| Total subjects, n | 251 | 246 | 236 | 178 | |  |
| No. of events | 50 | 37 | 27 | 17 | |  |
| HR (95% CI) | 1 | 0.58 (0.36-0.93) | 0.51 (0.30-0.88) | 0.48 (0.26-0.89) | | 0.01 |
| **c) Multivariate RR (95% CI) of incidence of fracture and butter consumption, stratified by gender.** | | | |  | |  |
|  | Butter (n, g/wk) | | | | | P-trend |
|  | 0≤n≤9.6 | 9.6<n≤20 | 20<n≤35 | 35<n | |  |
| Characteristics | **Men** | | | | |  |
| Total subjects, n | 182 | 185 | 214 | 254 | |  |
| No. of events | 41 | 39 | 30 | 40 | |  |
| HR (95% CI) | 1 | 0.90 (0.57-1.43) | 0.53 (0.33-0.87) | 0.67 (0.42-1.08) | | 0.03 |
|  | **Women** | | | | |  |
| Total subjects, n | 256 | 250 | 223 | 182 | |  |
| No. of events | 82 | 72 | 77 | 66 | |  |
| HR (95% CI) | 1 | 0.85 (0.60-1.19) | 1.19 (0.84-1.67) | 1.24 (0.85-1.81) | | 0.11 |

^1^ Values are hazard ratios (95 % CIs) derived by Cox proportional hazards regression models adjusted for BMI, food energy intake, alcohol consumption, education, smoking, physical activity, family history of MI, multivitamin, serum cholesterol, triglycerides, incidence of hypertension.
